# Artificial intelligence in the diagnosis of dental diseases on panoramic radiographs: a preliminary study

**DOI:** 10.1186/s12903-023-03027-6

**Published:** 2023-06-03

**Authors:** Junhua Zhu, Zhi Chen, Jing Zhao, Yueyuan Yu, Xiaojuan Li, Kangjian Shi, Fan Zhang, Feifei Yu, Keying Shi, Zhe Sun, Nengjie Lin, Yuanna Zheng

**Affiliations:** 1 School/Hospital of Stomatology, Zhejiang Chinese Medical University, Hangzhou, Zhejiang China; 2 College of Computer Science and Technology, Zhejiang University of Technology, Hangzhou, Zhejiang China

**Keywords:** Artificial intelligence (AI), Dental disease, Diagnosis, Panoramic radiograph, Preliminary reading

## Abstract

**Background:**

Artificial intelligence (AI) has been introduced to interpret the panoramic radiographs (PRs). The aim of this study was to develop an AI framework to diagnose multiple dental diseases on PRs, and to initially evaluate its performance.

**Methods:**

The AI framework was developed based on 2 deep convolutional neural networks (CNNs), BDU-Net and nnU-Net. 1996 PRs were used for training. Diagnostic evaluation was performed on a separate evaluation dataset including 282 PRs. Sensitivity, specificity, Youden’s index, the area under the curve (AUC), and diagnostic time were calculated. Dentists with 3 different levels of seniority (H: high, M: medium, L: low) diagnosed the same evaluation dataset independently. Mann-Whitney U test and Delong test were conducted for statistical analysis (ɑ=0.05).

**Results:**

Sensitivity, specificity, and Youden’s index of the framework for diagnosing 5 diseases were 0.964, 0.996, 0.960 (impacted teeth), 0.953, 0.998, 0.951 (full crowns), 0.871, 0.999, 0.870 (residual roots), 0.885, 0.994, 0.879 (missing teeth), and 0.554, 0.990, 0.544 (caries), respectively. AUC of the framework for the diseases were 0.980 (95%CI: 0.976–0.983, impacted teeth), 0.975 (95%CI: 0.972–0.978, full crowns), and 0.935 (95%CI: 0.929–0.940, residual roots), 0.939 (95%CI: 0.934–0.944, missing teeth), and 0.772 (95%CI: 0.764–0.781, caries), respectively. AUC of the AI framework was comparable to that of all dentists in diagnosing residual roots (p > 0.05), and its AUC values were similar to (p > 0.05) or better than (p < 0.05) that of M-level dentists for diagnosing 5 diseases. But AUC of the framework was statistically lower than some of H-level dentists for diagnosing impacted teeth, missing teeth, and caries (p < 0.05). The mean diagnostic time of the framework was significantly shorter than that of all dentists (p < 0.001).

**Conclusions:**

The AI framework based on BDU-Net and nnU-Net demonstrated high specificity on diagnosing impacted teeth, full crowns, missing teeth, residual roots, and caries with high efficiency. The clinical feasibility of AI framework was preliminary verified since its performance was similar to or even better than the dentists with 3–10 years of experience. However, the AI framework for caries diagnosis should be improved.

## Background

Dental diseases are prevalent all over the world. According to the 2017 Global Burden of Disease study, approximately 3.5 billion people worldwide suffer from dental diseases, mainly untreated caries, severe periodontal disease, edentulism, and severe tooth loss (with just 1 to 9 remaining teeth) [1]. Dental diseases, especially untreated ones, may cause infections, pain, restricted mouth opening and even life-threatening conditions that seriously affect quality of life, productivity and work capacity, and social participation of patients [2].

Clinical examination combined with radiographs is a commonly used method for the diagnosis of dental diseases [3]. Due to the complex anatomy and progress of diseases, interpreting radiographs quickly and accurately is challenging for the dentists [4]. Artificial intelligence (AI) have been proven to significantly increase the workflow efficiency and accuracy in the field of medical imaging [5]. Nowadays, images in dentistry are commonly digitizing and easily translated into computer language [6]. Therefore, the application of AI in the auxiliary diagnosis of dental diseases is promising [7, 8].

In the field of oral and maxillofacial radiology, the studies on the application of AI were mainly based on panoramic radiographs (PRs) [9], since they have a wide range of display, can be easily obtained in dental clinic, and are suitable for the computer-aid diagnose of various dental diseases or conditions [10]. However, low contrast, overlapping structures and unclear edges of teeth in PRs increase the difficulty of segmentation [11, 12]. In recent years, considerable results in the segmentation have been achieved by using of convolutional neural networks (CNNs)-based image segmentation [13, 14]. Many CNNs-based models are developed for the diagnosis of a particular disease or condition [15–19]. However, in fact, patients always suffer from multiple dental diseases at the same time, which can be identified by PRs [20, 21]. Until now, there are limited studies related to CNN-based diagnosis of multiple diseases on PRs [22–24].

In our previous study, we proposed a dual subnetworks structure based on border guidance and feature map distortion, called BDU-Net [25]. It showed great potential on improving the performance of teeth instance segmentation. In the presence of missing teeth or misalignment, BDU-Net’s segmentation performance appeared to be better than other networks. Therefore, in this study we aimed to built an AI framework based on 2 deep CNNs, BDU-Net and no-new-Net (nnU-Net) for diagnosing 5 common dental diseases on PRs, and the null hypothesis was that there is no difference between the performance of AI framework and dentists. The initial performance of the AI framework on diagnosing dental diseases was satisfactory, except caries. The clinical feasibility of the AI framework was preliminary verified by comparing with the diagnosing results and efficiency of dentists with different experience. But at the same time, some limitations and problems were revealed.

## Methods

### Ethics approval

The study was conducted at the Stomatology Hospital of Zhejiang Chinese Medical University. PRs were taken with the patients’ informed consents for their therapeutic or diagnostic purposes, and these data could be used for medical research without compromising their privacy. Therefore, no additional informed consents from these patients were added to this study. The study was approved by the Ethics Committee of Stomatology Hospital of Zhejiang Chinese Medical University (approval no. 330,108,002 − 202,200,005), and was performed in accordance with the Declaration of Helsinki.

### Selection of panoramic radiographs

The PRs were retrospectively selected from an image database of patients who visited the hospital between April 2019 and July 2021. The inclusion criteria for PRs included: permanent dentition: age > 16. The exclusion criteria included: (1) retained deciduous teeth and deciduous dentition; (2) severe crowded teeth (more than 8 mm per arch); (3) blurred and incomplete PRs were excluded from further analysis; (4) artifact of earrings, glasses and removable dentures on the PRs; (5) edentulous jaw. All PRs were produced using a Sirona digital machine (ORTHOPHOS XG 5 DS Ceph, Bensheim, Germany) with standard parameters, operating with tube voltages between 60 and 90 kV and tube operating currents between 1 and 10 mA. A default program of the device with a predetermined magnification of 1.9× and a rotation time of 14.1 s was used for X-ray exposures. The resolution of PRs was 2440 × 1280. PRs were exported to Portable Network Graphics (PNG) format.

### Annotation of the data

A total of 1996 images of 1996 patients including 912 males and 1084 females, with a mean age of 37 years (ranging from 17 ~ 83 years old) made up the training dataset. A free open-source software 3D Slicer was applied as the annotation tool. Three dentists with more than 12 years of clinical experience independently and blindly marked the areas of impacted teeth, residual roots, caries, full crowns, and other teeth on the PRs. All caries, which were identifiable on PRs, both primary and secondary, were marked. It meant that early caries that have not caused hard tissue defects were not studied. The annotated images were reviewed and revised by another 2 oral and maxillofacial imaging experts and achieved final confirmation [26]. Prior to the annotation and review process, each participant was instructed and calibrated on the annotation task using a standardized protocol. The set of common points of most labels was selected as ground truth.

All confirmed data were divided into 3 mutually exclusive sets. The training set in Table 1 was used to train the framework. The validation set was used in the training phase to verify the effectiveness of the framework training and to select hyperparameters. The test set was used for initial framework performance evaluation.

**Table 1:** The numbers of diseases in the training set and evaluation set

Diseases	training set	evaluation set
Caries	1648	689
Residual roots	230	62
Impacted teeth	808	384
Full crowns	256	275
Missing teeth	1091	512

### The AI framework development

**Fig. 1:**
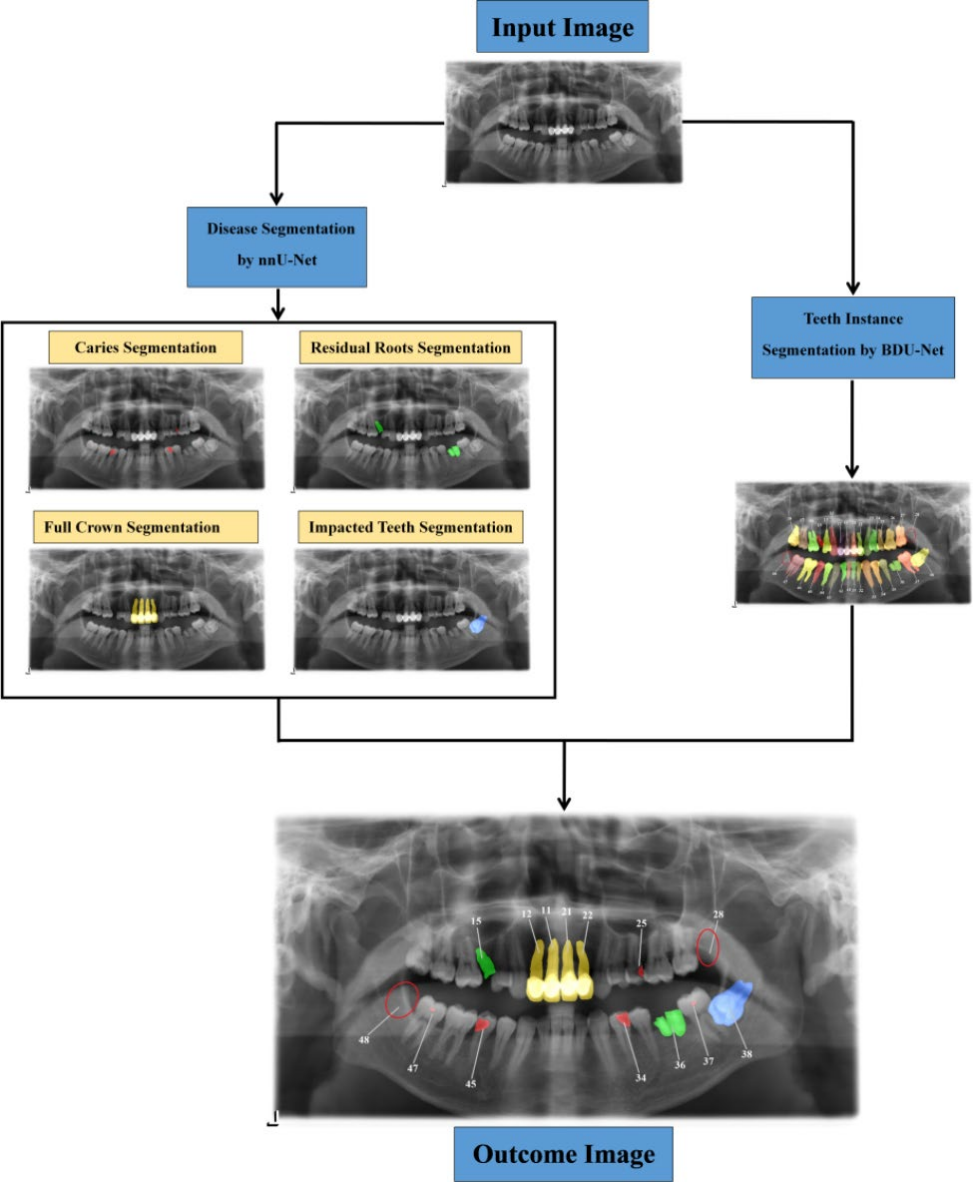
Multiple dental diseases’ diagnostic process of the proposed AI framework on PRs.

Our proposed AI framework incorporated a full-mouth teeth instance segmentation network and a multiple dental disease segmentation network to enable the diagnosis of multiple dental diseases on PRs within a single framework (Fig. 1).

**Fig. 2:**
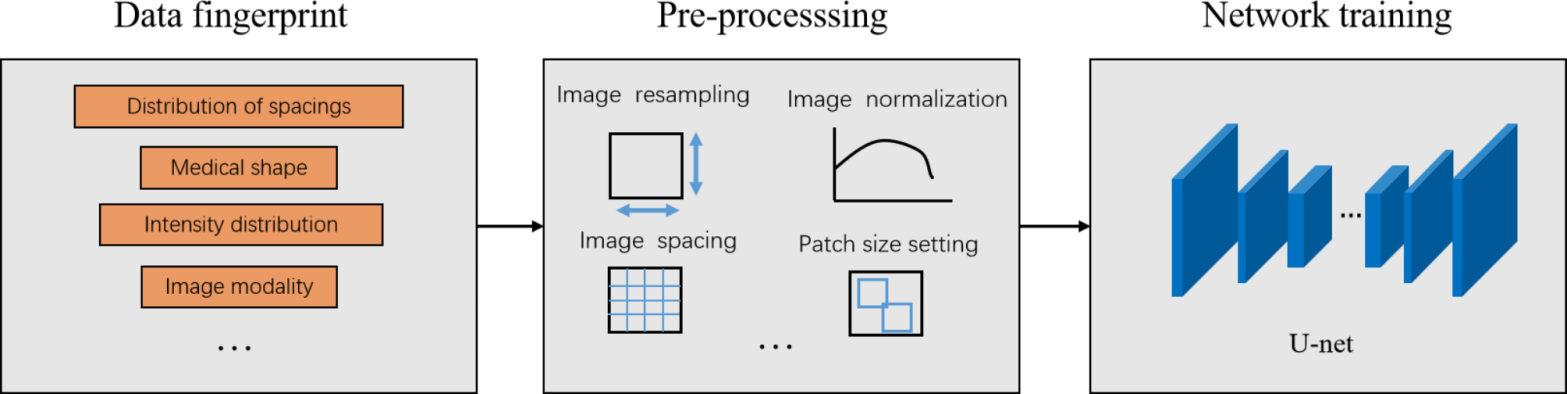
The workflow of disease segmentation by nnU-Net.

nnU-Net was used to segment the semantics of dental diseases. Since one nnU-Net can segment just one single disease, 4 parallel nnU-Net were designed for segment impacted teeth, residual roots, caries, and full crowns respectively. Like the other U-Net architectures, a U-shaped configuration of convolutional network layers with skip connections was designed [27]. nnU-Net analyzed the characteristics of the input dataset and performs suitable pre-processing operations on the dataset based on the information obtained from the analysis. The hyperparameters in nnU-Net was automatically set, such as training batch size, image block size, down-sampling times, etc. (Fig. 2). This study used a five-fold cross-validation approach, using cross-entropy loss and dice loss as loss functions during the training process. We chose Adam as the optimizer, with the learning rate set to a dynamic adjustment strategy and used an online data augmentation strategy during the training process.

**Fig. 3:**
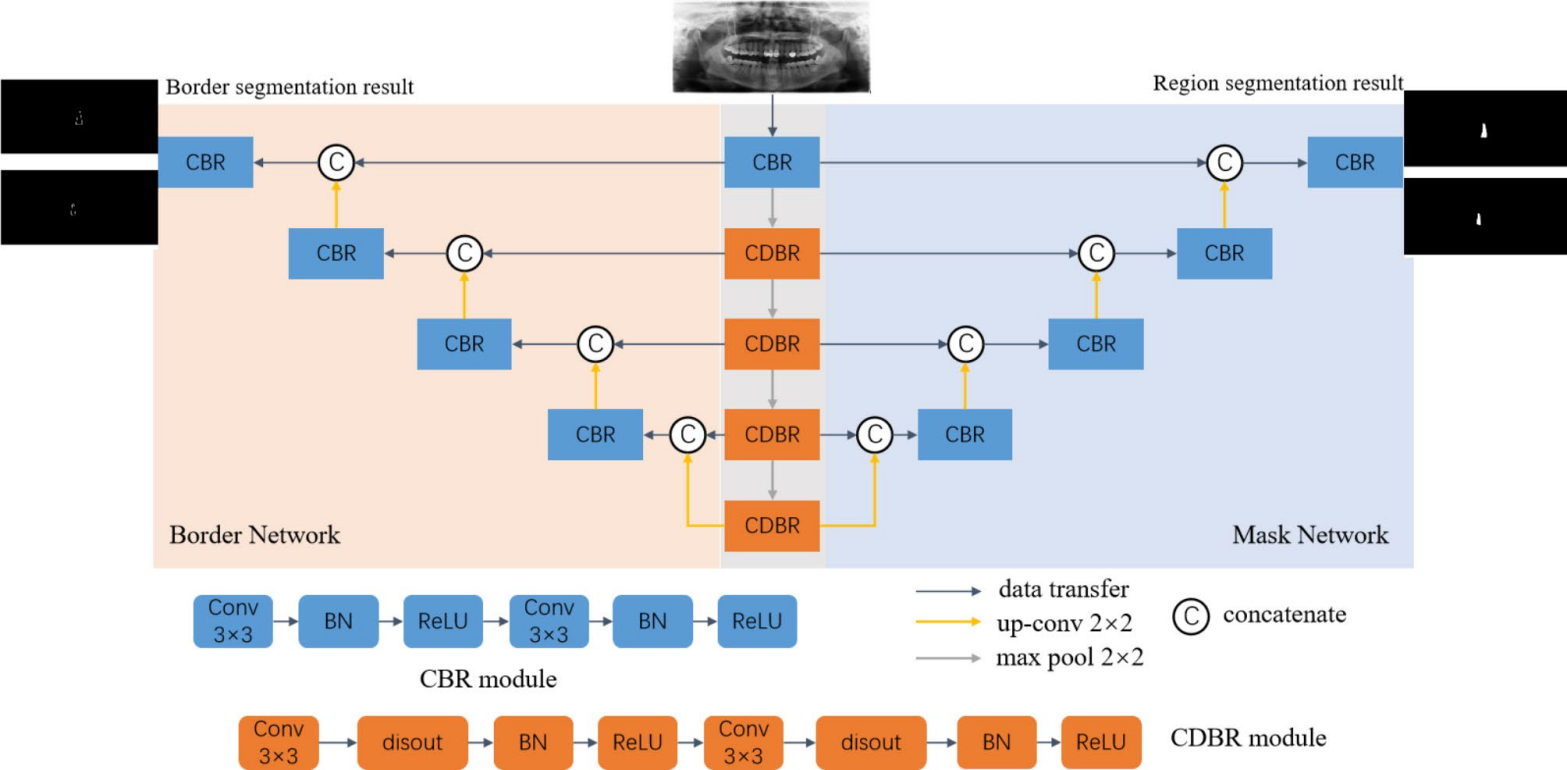
The structure of BDU-Net for teeth instance segmentation

In order to obtain the tooth position information and further diagnose the missing teeth, teeth instance segmentation network called BDU-Net was introduced. BDU-Net is mainly composed of two sub-networks. One is the region sub-network used to generate the region segmentation results, and the other is the border sub-network that adjusts the segmentation boundaries (Fig. 3). In this study, BDU-Net was used to segment all the teeth on the PRs. Teeth were numbering and the missing teeth were reported. We generated boundary labels using the Canny algorithm based on conventional boundary detection, which did not rely on additional manual annotation [28]. During training, random affine elastic transformation was used to augment the data. To ensure fairness, all experiments were implemented with the SGD optimizer, where the learning rate was 0.01, the momentum was 0.9, the batch size was 1, and the number of epochs was 100. The network was implemented on NVIDIA GeForce RTX 2080Ti GPU using PyTorch framework. Finally, the 2 segmentation results were combined, and a complete complementary diagnostic result with both disease type and disease location was generated.

The sensitivity and specificity of the AI framework for the detection of 5 different dental diseases were initially evaluated by using test set. Sensitivity (Sen) refers to the ability of the framework to find all positive samples, that is, how many real positive samples can be covered by the prediction results given by the framework. Similarly, specificity (Spe) is used for negative samples, that is, how many of the actual negative samples are predicted correctly. The index values were calculated using confusion matrix. The sensitivity and specificity were calculated according to the following formula:

$$S{\text{en}}=\frac{{TP}}{{TP+FN}}$$

$$Spe=\frac{{TN}}{{TN+FP}}$$

TP, TN, FP and FN denote true positives, true negatives, false positives and false negatives, respectively.

The results of sensitivity and specificity were 0.863 and 0.983 for diagnosing missing teeth, 0.821 and 0.989 for diagnosing caries, 0.718 and 0.997 for diagnosing residual roots, 0.942 and 0.986 for diagnosing impacted teeth, and 0.835 and 0.991 for diagnosing full crowns. These results were close to or better than relevant studies [24, 29].

### Separate evaluation dataset

The diagnostic performance of the proposed framework was evaluated by using a separate evaluation dataset. The sample size of the dataset was calculated according to the following formula:

$${n_{Sen}}=\frac{{Z_{{\frac{\alpha }{2}}}^{2}Sen{\text{(1-}}Sen{\text{)}}}}{{{d^2} \times Prev}}$$

$${n_{Spe}}=\frac{{Z_{{\frac{\alpha }{2}}}^{2}Spe{\text{(1-}}Spe{\text{)}}}}{{{d^2} \times (1 - Prev)}}$$

Prev is prevalence, d means the precision of estimate (i.e. the maximum marginal error) [30]. According to the literature, the Prev for these 5 diseases were set as 86.2%, 60.37%, 24.6%, 24%, and 22.3%, respectively [31–34]. For ɑ is 0.05, ${Z}_{\frac{\text{?}}{2}}$ is inserted by 1.96, and d is 0.1. The sample size calculated using the above parameters was N(max)= (53, 94, 226, 95, 221, 11, 2, 7, 5, 47). Therefore, the recommended sample size was 226. In the present study, a total of 282 images of 282 patients including 131 males and 151 females, with a mean age of 34 years (ranging from 18 ~ 85 years old), made up the final evaluation dataset.

Three dentists, each with more than 15 years of experience and who did not attend the annotation of the previous training dataset, independently read the images and made diagnoses. Any disagreements were discussed among all three dentists, and consensus results were used as the gold standard.

### Performance evaluation of the proposed AI framework

The 282 PRs were uploaded to the framework and automatically read and marked. Since the images lacked annotations, classification indicators were used to assess the dentists’ performance and the framework’s performance, instead of segmentation indicators. Sensitivity, specificity, Youden’s index, and AUC were assessed. Youden’s index was calculated according to the formula:

$$Youden'\operatorname{s} {\text{ }}index = Sen + Spe - 1$$

AUC is an effective way to summarize the overall diagnostic accuracy of the test, which was calculated by MedCalc Statistical Software version 19.2.1 (MedCalc Software Ltd., Ostend, Belgium).

To test the validity of the framework, 9 dentists with 3 different levels of seniority from the Stomatology Hospital of Zhejiang Chinese Medical University were invited to evaluate the same batch of PRs independently, and to generate clinical imaging report of each PR. Three dentists with high seniority had over 10 years of clinical experience (H1, H2, H3), 3 dentists with medium seniority had 3–10 years of clinical experience (M1, M2, M3), and 3 dentists with low seniority had less than 3 years of clinical experience (L1, L2, L3). Before starting the experiment, dentists were pre-trained to diagnose 5 dental diseases on PRs to familiarize themselves with the pattern of diagnosis. The diagnostic results of 5 diseases from 9 dentists and the framework were compared with the gold standard (Table 1).

Diagnostic time of both the framework and the dentists was calculated to evaluate the efficiency. The framework’s diagnostic time was the time taken from the image input to the result output, which was recorded automatically on the computer. The dentists’ diagnostic time was measured by an observer using a stopwatch, starting when the image was opened on the computer and ending when the dentist had completed the initial full diagnosis of the PR.

### Statistical analysis

Mann-Whitney U test was used to assess the differences between the diagnostic time of framework and dentists. Statistical analysis was conducted using the SPSS 26.0 software (IBM SPSS Statistics Base Integrated Edition 26, Armonk, NY, USA). The results of AUC of the framework and the dentists were statistical analyzed in MedCalc Statistical Software version 19.2.1 (MedCalc Software Ltd., Ostend, Belgium) by using the DeLong test. The statistical levels of significance were both set at ɑ=0.05.

## Results

Table 2 shows the diagnostic performance of the framework for impacted teeth. Compared with dentists, the framework had the lowest specificity (0.996). The framework’s sensitivity (0.964), Youden’s index (0.960), and AUC (0.980) were similar to M3, and just lower than that of H1 and H2. The AUC of the framework was significantly higher than M1, M2, L1, L2, and L3 (p < 0.05), and significantly lower than H1 (p < 0.05).

**Table 2:** Diagnostic performance of the framework and individual dentists for impacted teeth

Var	Spe	Sen	Youden’s index	AUC (SE; 95% CI)	P	Z
Framework	0.996	0.964	0.960	0.980 (0.00480; 0.976–0.983)	ref	ref
H1	0.997	0.987	0.984	0.992 (0.00291; 0.990–0.994)	0.009*	2.629
H2	0.998	0.966	0.964	0.982 (0.00463; 0.979–0.985)	0.607	0.514
H3	0.997	0.956	0.953	0.976 (0.00527; 0.973–0.980)	0.501	0.673
M1	0.998	0.924	0.922	0.961 (0.00676; 0.957–0.966)	0.013*	2.487
M2	0.999	0.932	0.931	0.966 (0.00642; 0.962–0.970)	0.049*	1.972
M3	0.997	0.964	0.961	0.980 (0.00480; 0.977–0.983)	0.906	0.118
L1	0.999	0.878	0.877	0.938 (0.00837; 0.933–0.944)	< 0.0001*	5.009
L2	0.999	0.901	0.900	0.950 (0.00763; 0.945–0.955)	0.000*	3.876
L3	1.000	0.883	0.883	0.941 (0.00822; 0.936–0.947)	< 0.0001*	4.531

Delong test was conducted for statistically analysis of the difference of AUC between the framework and the dentists. * represents significant difference (p < 0.05)

Table 3 shows the diagnostic performance of the framework for full crowns. Compared with dentists, the framework had the lowest specificity (0.998). The framework’s sensitivity (0.953), Youden’s index (0.951), and AUC (0.975) were at medium level, which were lower than those of all H-level dentists. The significant difference of AUC only existed between the framework and L2 (p < 0.05).

**Table 3:** Diagnostic performance of the framework and individual dentists for full crowns

Var	Spe	Sen	Youden’s index	AUC (SE; 95% CI)	P	Z
Framework	0.998	0.953	0.951	0.975 (0.00641; 0.972–0.978)	ref	ref
H1	0.999	0.971	0.970	0.985 (0.00508; 0.982–0.988)	0.241	1.173
H2	0.999	0.960	0.959	0.980 (0.00592; 0.976–0.982)	0.642	0.465
H3	1.000	0.964	0.964	0.982 (0.00566; 0.979–0.984)	0.458	0.743
M1	0.999	0.953	0.952	0.976 (0.00641; 0.973–0.979)	0.944	0.070
M2	1.000	0.938	0.938	0.969 (0.00728; 0.965–0.972)	0.464	0.732
M3	1.000	0.953	0.953	0.976 (0.00641; 0.973–0.979)	0.922	0.098
L1	0.999	0.920	0.919	0.960 (0.00820; 0.955–0.964)	0.130	1.516
L2	0.999	0.909	0.908	0.954 (0.00869; 0.950–0.958)	0.037*	2.090
L3	0.999	0.956	0.955	0.978 (0.00617; 0.974–0.981)	0.775	0.286

Delong test was conducted for statistically analysis of the difference of AUC between the framework and the dentists. * represents significant difference (p < 0.05)

Table 4 shows the diagnostic performance of the framework for missing teeth. Compared with dentists, the framework had the lowest specificity (0.994). The framework’s sensitivity (0.885), Youden’s index (0.879), and AUC (0.939) were at medium level, which were lower than those of all H-level dentists. The AUC of the framework was significantly lower than H2 and H3 (p < 0.05), and was significantly higher than L1 (p < 0.05).

**Table 4:** Diagnostic performance of the framework and individual dentists for missing teeth

Var	Spe	Sen	Youden’s index	AUC (SE; 95% CI)	P	Z
Framework	0.994	0.885	0.879	0.939 (0.00708; 0.934–0.944)	ref	ref
H1	0.995	0.889	0.884	0.942 (0.00697; 0.937–0.947)	0.748	0.321
H2	0.998	0.914	0.912	0.956 (0.00620; 0.951–0.960)	0.023*	2.279
H3	0.997	0.920	0.917	0.958 (0.00601; 0.954–0.963)	0.012*	2.515
M1	0.997	0.885	0.882	0.941 (0.00707; 0.935–0.946)	0.857	0.180
M2	0.996	0.900	0.896	0.948 (0.00663; 0.943–0.953)	0.268	1.109
M3	0.996	0.869	0.865	0.933 (0.00747; 0.927–0.938)	0.448	0.759
L1	0.994	0.832	0.826	0.913 (0.00828; 0.907–0.919)	0.006*	2.770
L2	0.997	0.875	0.872	0.936 (0.00732; 0.931–0.941)	0.707	0.376
L3	0.996	0.852	0.848	0.924 (0.00787; 0.918–0.930)	0.053	1.938

Delong test was conducted for statistically analysis of the difference of AUC between the framework and the dentists. * represents significant difference (p < 0.05)

Table 5 shows the diagnostic performance of the framework for residual roots. The specificity of the framework (0.999) was very close or equal to that of all dentists. The framework’s sensitivity (0.871), Youden’s index (0.870), and AUC (0.935) were at medium level, which were lower than those of all H-level dentists. No significant difference of the AUC was found between the framework and dentists (p > 0.05).

**Table 5:** Diagnostic performance of the framework and individual dentists for residual roots

Var	Spe	Sen	Youden’s index	AUC (SE; 95% CI)	P	Z
Framework	0.999	0.871	0.870	0.935 (0.02150; 0.929–0.940)	ref	ref
H1	1.000	0.919	0.919	0.960 (0.01740; 0.955–0.964)	0.310	1.016
H2	0.999	0.919	0.918	0.959 (0.01740; 0.955–0.963)	0.316	1.003
H3	1.000	0.887	0.887	0.943 (0.02030; 0.938–0.948)	0.731	0.344
M1	0.999	0.823	0.822	0.911 (0.02450; 0.905–0.917)	0.412	0.821
M2	0.999	0.839	0.838	0.919 (0.02350; 0.913–0.925)	0.567	0.572
M3	0.999	0.855	0.854	0.927 (0.02260; 0.921–0.932)	0.761	0.304
L1	0.999	0.839	0.838	0.919 (0.02350; 0.912–0.925)	0.592	0.536
L2	1.000	0.871	0.871	0.935 (0.02150; 0.930–0.941)	0.987	0.017
L3	0.999	0.839	0.838	0.919 (0.02350; 0.913–0.925)	0.569	0.570

Delong test was conducted for statistically analysis of the difference of AUC between the framework and the dentists. * represents significant difference (p < 0.05)

Table 6 shows the diagnostic performance of the framework for caries. Compared with dentists, the framework had the highest specificity (0.990). The framework’s sensitivity (0.554), Youden’s index (0.544), and AUC ( 0.772) were nearly lower than that of all dentists in M- and H-level. The AUC of the framework was significantly lower than H1, H2, and H3 (p < 0.05), and significantly higher than L1 and L3 (p < 0.05).

**Table 6:** Diagnostic performance of the framework and individual dentists for caries

Var	Spe	Sen	Youden’s index	AUC (SE; 95% CI)	P	Z
Framework	0.990	0.554	0.544	0.772 (0.00949; 0.764–0.781)	ref	ref
H1	0.980	0.621	0.601	0.800 (0.00928; 0.792–0.809)	0.007*	2.721
H2	0.944	0.755	0.699	0.849 (0.00830; 0.842–0.857)	< 0.0001*	7.099
H3	0.975	0.669	0.644	0.822 (0.00901;0.814–0.830)	< 0.0001*	4.799
M1	0.978	0.569	0.547	0.773 (0.00947; 0.765–0.782)	0.917	0.104
M2	0.976	0.586	0.562	0.781 (0.00943;0.772–0.789)	0.424	0.800
M3	0.988	0.546	0.534	0.767 (0.00951; 0.758–0.775)	0.623	0.492
L1	0.988	0.417	0.405	0.702 (0.00942; 0.693–0.712)	< 0.0001*	6.847
L2	0.995	0.509	0.504	0.752 (0.00954; 0.743–0.761)	0.060	1.884
L3	0.990	0.402	0.392	0.696 (0.00936; 0.687–0.706)	< 0.0001*	6.859

Delong test was conducted for statistically analysis of the difference of AUC between the framework and the dentists. * represents significant difference (p < 0.05)

**Fig. 4:**
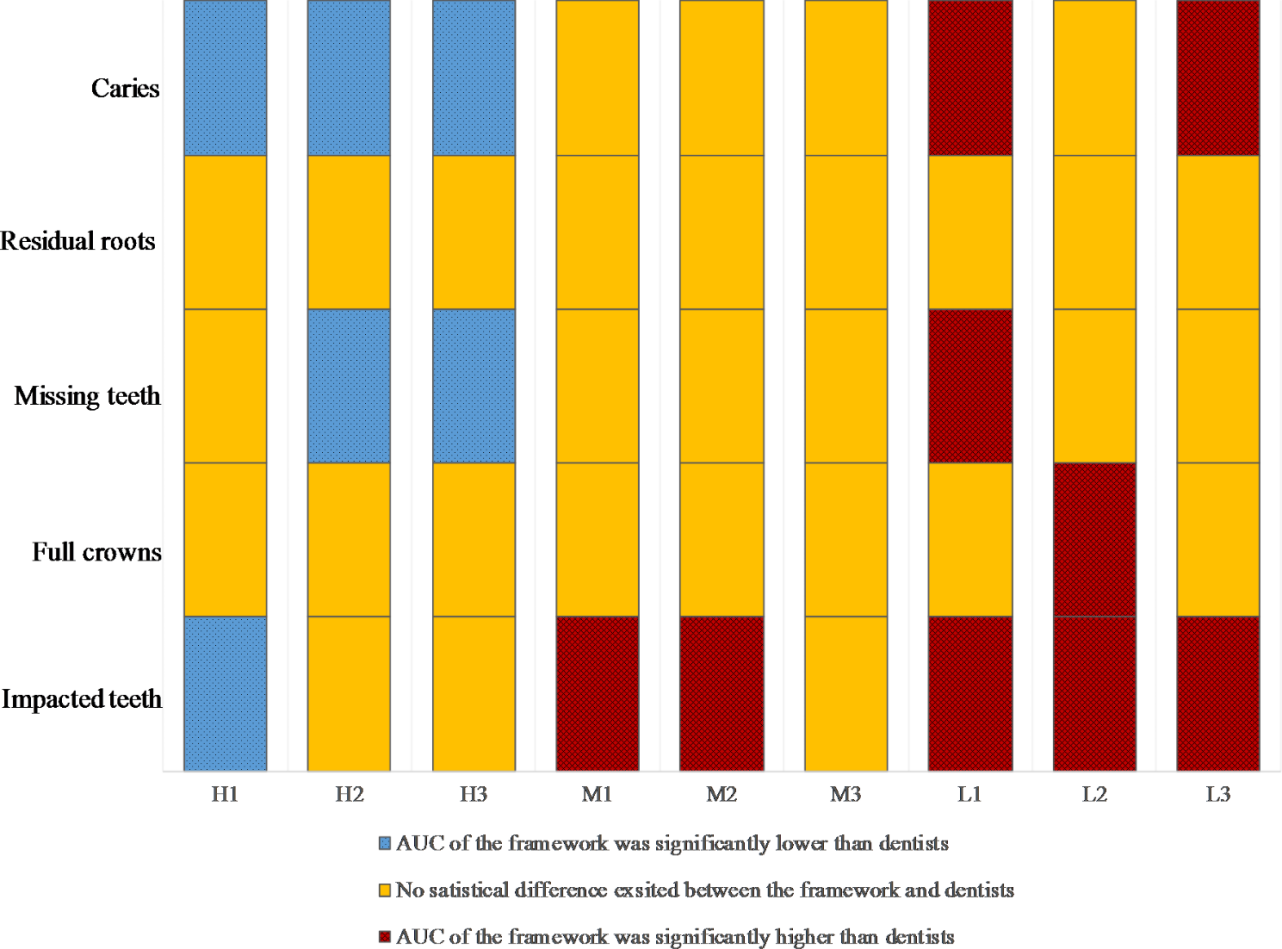
Schematic representation of Delong test results based on AUC for 5 different diseases. The results of the framework were compared with 9 dentists at 3 different levels of seniority (H: high, M: medium, L: low), and statistically significant difference was set as p < 0.05

The Delong tests for the statistical analysis of AUC between the framework and dentists are summarized in Fig. 4. The framework exhibited performance comparable or even better than the M-level dentists on diagnosing dental diseases. Especially on diagnosing residual roots and full crowns, the framework’s performance reached the same level as that of H-level dentists.

**Fig. 5:**
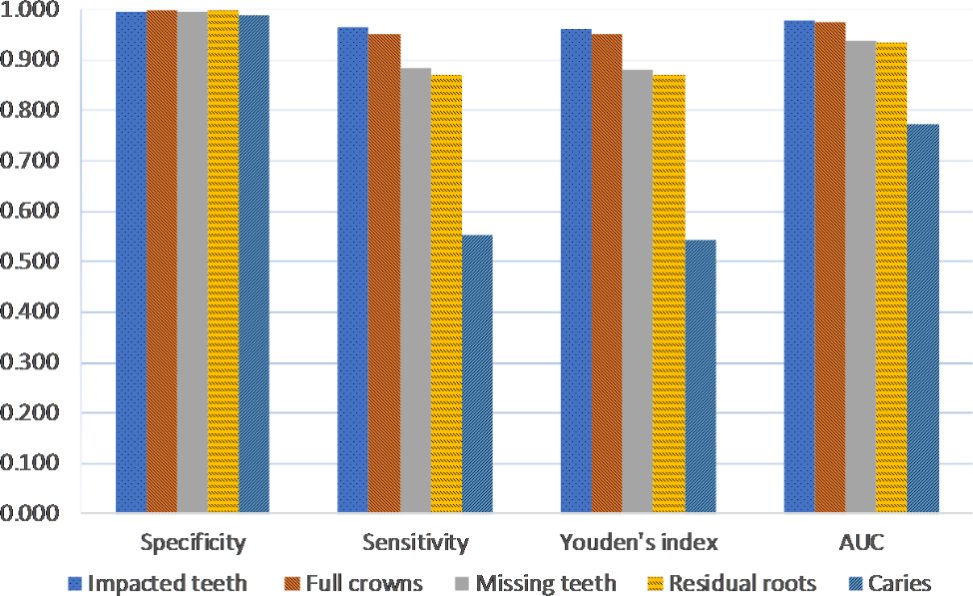
Performance of the proposed AI framework on 5 diseases based on 4 evaluation indexes

The comparison of the framework’s performance on diagnosing 5 different diseases are shown in Fig. 5. For both impacted teeth and full crowns, all 4 indexes of the framework were over 0.95. The framework’s specificity for diagnosing 5 diseases were as high as 0.99 and above. However, the range of the other 3 indexes when the framework diagnosed different diseases was very large. Among 5 diseases, the framework achieved the highest sensitivity, Youden’s index, and AUC in diagnosing impacted teeth, and the highest specificity in diagnosing residual roots. Meanwhile, the lowest sensitivity, specificity, Youden’s index, and AUC of the framework were obtained in diagnosing caries, which were consisted with the results of dentists.

The mean diagnostic time of the framework per PR (1.5 ± 0.3 s) was about 35 times shorter than that of all dentists (53.8 ± 46.0 s), in Table 7. The mean diagnostic time of the framework was significantly lower than that of all dentists (p < 0.001).

**Table 7:** Mean time for initial interpretation of each panoramic radiograph

Groups	Mean time (s)		
	Mean time per dentist	Mean time for 3 levels of dentists	Mean time for all dentists
Framework	1.5 ± 0.3*		
H1	30.0 ± 33.4*	37.2 ± 27.9	53.8 ± 46.0
H2	41.7 ± 22.4*		
H3	39.9 ± 25.5*		
M1	50.7 ± 27.6*	52.1 ± 38.2	
M2	48.5 ± 26.9*		
M3	57.0 ± 53.6*		
L1	94.0 ± 76.0*	72.0 ± 59.3	
L2	67.0 ± 55.8*		
L3	55.0 ± 29.4*		

* represents mean time of the framework was significantly lower than that of all dentists (p < 0.001).

## Discussion

In order to improve the efficiency of interpreting PRs, reduce misdiagnosis, and mitigate missed diagnoses caused by human factors, we proposed an AI framework for diagnosing multiple dental diseases on PRs. The null hypothesis of this study was rejected, as the difference in diagnostic performance existed between AI and dentists with different levels of seniority.

The U-net network structure, which combines the deep semantic information and shallow image detail information of neural network, performed well on medical image segmentation. In this study, we jointly applied two improved versions of U-net, namely nnU-Net and BDU-Net, to build an AI framework for the first time. Selecting a suitable network for each disease separately would be too cumbersome and not conducive to subsequent extension of the framework to other diseases. nnU-Net can automatically adapt to any dataset by adjusting the hyperparameters according to the data characteristics [35]. BDU-Net focuses on enhanced generalization capabilities and instance boundary adjustment, improving not only the accuracy of tooth position identification, but also achieving more accurate segmentation results for teeth boundaries [25].

Previous studies on diagnosing multiple dental diseases by AI have been limited. Zadrozny et al. [23] evaluated the reliability of a commercial AI model for detecting multiple conditions on 30 PRs. The specificities were over 0.9 except for detecting periodontal bone loss, but the sensitivity of the model for detecting different conditions varied greatly. The 2 highest sensitivities were 0.961 for missing teeth and 0.957 for restorations, and the 2 lowest sensitivities were 0.445 for caries and 0.390 for periapical lesion. Basaran et al. [24] evaluated the performance of another commercial AI model based on Faster R-CNN method and Google Net Inception v2 architecture. A large evaluation dataset, including 1084 PRs, was used to detect 10 conditions, with the sensitivity of the model ranging from 0.3026 ~ 0.9674, the precision ranging from 0.1923 ~ 0.9259, and the F1 score ranging from 0.1257 ~ 0.9433, respectively. The results of these 3 indexes were consistent, indicating that the model performed well in detecting crowns, implants, and fillings, but faced challenges in accurately detecting caries and dental calculus. Vinayahalingam et al. [22] developed a new model based on mask R-CNN with Resnet-50 in combination with a rule-based heuristic algorithm and a combinatorial search algorithm. The model was trained on 2000 PRs, with 200 of them set as a test dataset. The precision, recall, and F1 score of the model for detecting teeth, crowns, implants, fillings, and root canal fillings were all above 0.90, but for root remnant, they were 0.852, 0.766, and 0.807, respectively.

In this study, the diagnostic performance of the AI framework was evaluated using a separate evaluation dataset and compared with dentists of different experience levels. Both the framework and the dentists demonstrated high specificity in diagnosing the five diseases, with the framework’s performance being particularly stable, exhibiting a specificity of 0.99 or higher. This suggests that the framework had very small prediction errors, effectively controlling false positives [22]. The framework was able to filter out most teeth that did not contain diseases, reducing the examination burden on dentists. Consistent with previous research, the framework performed better than dentists in terms of sensitivity, screening performance, and overall diagnostic accuracy in the diagnosis of impacted teeth and crowns. These conditions had high contrast and clear boundaries on PRs, making them easy to distinguish. However, for missing teeth and residual roots, while the AUC values were generally high, the sensitivity and Youden’s index were lower than 0.90. This indicated that non-detection errors existed in the framework, such as residual roots being mistaken for teeth, and second molars being identified as third molars [22]. Additionally, for caries, the sensitivity and Youden index decreased further, falling below 0.6. This could be due to the significant variation in the position, extent, and shape of caries. Some caries, such as interproximal dish-shaped root caries and caries with smaller cavitary changes, are not easy to be detected in the clinic without X-ray test. As a result, the structures would be under-segmented by the framework, leading to an increase in false negatives [22]. Previous research on clinical visual inspection of caries showed that the sensitivity of dentists to detect occlusal caries (0.777) was significantly higher than that of proximal caries (0.224) [36], which is consistent with the results of the dentists and framework in this study. Efforts have been made to improve the accuracy of AI for detecting caries, such as the application of a gradient-weighted class activation map in MobileNet V2 to highlight carious areas in cropped images for the classification of caries lesions [37]. These findings provide ideas for adjusting the framework in the future.

Overall, the framework demonstrated similar or better overall diagnostic accuracy than M-level dentists and, in many cases, outperformed L-level dentists. In the diagnosis of residual roots and crowns, the framework’s overall diagnostic accuracy reached the level of H-level dentists. However, for caries, H-level dentists performed significantly better than the framework. Additionally, the framework had clear advantages over dentists in terms of diagnostic efficiency, and there is still potential for further improvement.

AI can be utilized in various fields of dentistry to aid in diagnosis, treatment planning, and prediction of treatment outcomes [38]. In recent years, digital X-rays have greatly advanced the development of AI in dentistry [39, 40]. The large number of oral X-rays taken each year during routine dental practice provides a valuable resource for image interpretation and image-based diagnosis. In the future, AI will revolutionize clinical workflows. Taking AI reports based on digital images as an example, on one hand, patients can conveniently manage their oral health, and on the other hand, these reports can help dentists complete clinical examinations and diagnoses more efficiently and accurately [9]. Dentists must therefore possess the ability to critically evaluate and ethically use AI applications. To prepare for future changes, dental education must also evolve. Basic knowledge of AI should become an integral part of the theoretical curriculum. Moreover, students should be trained in scenarios that AI has already affected, such as patient communication and management, paper writing, etc. [41].

It is important to consider the limitations of this study, particularly regarding the interpretation of the results. First, the study only examined five common dental diseases, which may have impacted the generalizability of the findings. Additionally, images lack clinical data, and the diagnoses of diseases have not been clinically verified, which may result in machine learning-specific bias and overdiagnosis. When assessing performance, the lack of clinical examination in developing the gold standard may affect the reliability and generalizability of the results. In the future, more dental diseases will be included for training and developing the framework, such as cysts, fillings, and periodontal diseases, and we will attempt to combine PRs with electronic medical records for more rigorous model training and more accurate evaluation results [42]. Second, the limited number of images in this study were obtained from a single source, which may raise concerns about overfitting and generalization of the results. Therefore, it is imperative to construct a large, heterogeneous, multicenter panoptic slice dataset to ensure that all relevant changes in patient demographics and target patient disease status in the clinical setting are fully represented in the application of the system [43, 44]. Third, although the performance of the framework was compared with the results of clinical dentists, the clinical relevance still needs to be further improved. In the future, the framework’s impact on treatment decisions and patient outcomes will be implemented.

## Conclusions

The AI framework based on nnU-Net and BDU-Net was successfully developed, and demonstrated high efficiency and specificity on diagnosing impacted teeth, full crowns, missing teeth, residual roots, and caries. The clinical feasibility of AI framework was preliminary verified since its accuracy and efficiency was similar to or even better than the dentists with 3–10 years of experience. It indicated that the AI framework could improve the accuracy and speed of dental disease diagnosis and treatment planning, potentially leading to better patient outcomes and lower healthcare costs. Caries diagnosis by the AI framework remained a challenge, using AI for other dental imaging modalities or exploring ways to improve the accuracy of caries detection should be considered in the future study.

## Data Availability

The data presented in this study are available on request from the corresponding author. The data are not publicly available due to privacy restrictions.

## Abbreviations

AI: Artificial intelligence
PRs: panoramic radiographs
ROC: receiver operating characteristic
AUC: area under the ROC curve
CNNs: convolutional neural networks
PNG: Portable Network Graphics.
